# Hytrosavirus genetic diversity and eco-regional spread in *Glossina* species

**DOI:** 10.1186/s12866-018-1297-2

**Published:** 2018-11-23

**Authors:** Irene K. Meki, Henry M. Kariithi, Mehrdad Ahmadi, Andrew G. Parker, Marc J. B. Vreysen, Just M. Vlak, Monique M. van Oers, Adly M.M. Abd-Alla

**Affiliations:** 1 Insect Pest Control Laboratory, Joint FAO/IAEA Programme of Nuclear Techniques in Food and Agriculture, International Atomic Energy Agency, Vienna International Centre, P.O. Box 100 1400, Vienna, Austria; 2 Laboratory of Virology, Wageningen University and Research, 6708 PB Wageningen, The Netherlands; 3 Biotechnology Research Institute, Kenya Agricultural and Livestock Research Organization, P.O Box 57811, Loresho, Nairobi, Kenya; 4 Insect Genetics Unit, Nuclear Science and Technology Research Institute, Karaj, Iran

**Keywords:** Salivary gland hypertrophy virus, GpSGHV, Genetic diversity, Sterile insect technique, Glossinidae, Tsetse, Virus evolution, Haplotype

## Abstract

**Background:**

The management of the tsetse species *Glossina pallidipes* (Diptera; Glossinidae) in Africa by the sterile insect technique (SIT) has been hindered by infections of *G. pallidipes* production colonies with *Glossina pallidipes* salivary gland hypertrophy virus (GpSGHV; *Hytrosaviridae* family). This virus can significantly decrease productivity of the *G. pallidipes* colonies. Here, we used three highly diverged genes and two variable number tandem repeat regions (VNTRs) of the GpSGHV genome to identify the viral haplotypes in seven *Glossina* species obtained from 29 African locations and determine their phylogenetic relatedness.

**Results:**

GpSGHV was detected in all analysed *Glossina* species using PCR. The highest GpSGHV prevalence was found in *G. pallidipes* colonized at FAO/IAEA Insect Pest Control Laboratory (IPCL) that originated from Uganda (100%) and Tanzania (88%), and a lower prevalence in *G. morsitans morsitans* from Tanzania (58%) and Zimbabwe (20%). Whereas GpSGHV was detected in 25–40% of *G. fuscipes fuscipes* in eastern Uganda, the virus was not detected in specimens of neighboring western Kenya. Most of the identified 15 haplotypes were restricted to specific *Glossina* species in distinct locations. Seven haplotypes were found exclusively in *G. pallidipes*. The reference haplotype H1 (GpSGHV-Uga; Ugandan strain) was the most widely distributed, but was not found in *G. swynnertoni* GpSGHV. The 15 haplotypes clustered into three distinct phylogenetic clades, the largest contained seven haplotypes, which were detected in six *Glossina* species. The *G. pallidipes*-infecting haplotypes H10, H11 and H12 (from Kenya) clustered with H7 (from Ethiopia), which presumably corresponds to the recently sequenced GpSGHV-Eth (Ethiopian) strain. These four haplotypes diverged the most from the reference H1 (GpSGHV-Uga). Haplotypes H1, H5 and H14 formed three main genealogy hubs, potentially representing the ancestors of the 15 haplotypes.

**Conclusion:**

These data identify G. pallidipes as a significant driver for the generation and diversity of GpSGHV variants. This information may provide control guidance when new tsetse colonies are established and hence, for improved management of the virus in tsetse rearing facilities that maintain multiple Glossina species.

**Electronic supplementary material:**

The online version of this article (10.1186/s12866-018-1297-2) contains supplementary material, which is available to authorized users.

## Background

Management of insect vectors using the sterile insect technique (SIT) within the context of area-wide integrated pest management (AW-IPM) approaches, requires mass-production of high quality insects that must outcompete wild males for mating virgin wild females [1]. These non-viable matings eventually lead to the decline of the target insect population and reduction in the occurrence of the trypanosomosis disease they transmit to animals and human. The successful eradication of a population of the tsetse fly species *Glossina austeni* Newstead (Diptera; Glossinidae) on Unguja Island in Zanzibar, using an AW-IPM approach with an SIT component [2] elicited efforts to apply a similar approach to eradicate *G. pallidipes* from the Southern Rift Valley region of Ethiopia [3]. However, like in many insect mass-production facilities where viral diseases challenge production of high quality insects [4], infections of *G. pallidipes* colonies with the *Glossina pallidipes* salivary gland hypertrophy virus (GpSGHV; *Hytrosaviridae* family) hindered full implementation of the SIT component of the Ethiopian program [5, 6].

The inbreeding in long-term colonized insects reduces their genetic diversity, i.e. fly populations become genetically more and more homozygous [7], which in return may promote their susceptibility to pathogen infections. Transmission, mainly horizontal, is further stimulated by the close interactions of tsetse conspecifics, the membrane feeding regimes, and the conducive environments created by the high densities in tsetse mass-production facilities [8]. Altogether these factors result in life-history trade-offs between immune and reproductive functions, which in turn contribute to reduced fitness-related traits of individual insects and colony productivity at large [9, 10]. In nature, where tsetse flies blood-feed on live animals, the observed GpSGHV infections are largely asymptomatic. This could be due to the low population densities and solitary habitats of different tsetse species, which underscores the triggering of SGH in the field or simply due to the short life span of symptomatically infected flies which minimizes the chances of detecting the SGH. [6, 11–13].

Currently, two GpSGHV strains induce distinctive pathologies in *G. pallidipes* flies, one in the mass-rearing facility at Kaliti in Ethiopia (Ethiopian strain with high SGH prevalence), and the other at the FAO/IAEA Insect Pest Control Laboratory (IPCL) in Seibersdorf, Austria (Ugandan strain with low SGH prevalence) [11]. It is not known why GpSGHV infections have such a devastating impact only on colonized *G. pallidipes*, despite the rearing of this species together with multiple *Glossina* species in the same tsetse production facilities. Kariithi et al., [14] reported that GpSGHV-infected *G. m. morsitans* expressed more antiviral proteins than symptomatically infected *G. pallidipes* flies. For example, the reactive oxygen species and components of the phagocytic engulfment system were among the overexpressed proteins in *G. m. morsitans*. The expression of such antiviral proteins in *G M. morsitans* and the reduced expression these genes in symptomatically infected *G. pallidipes* indicates that this *G. pallipides* is immunocompromised in its response to GpSGHV. Being the most susceptible species to GpSGHV, one may hypothesize *G. pallidipes* as a key species that drives the evolution and the inter-species spread of GpSGHV in tsetse mass rearing facilities.

To manage the prevalence of SGH in tsetse mass-production facilities, it is necessary to understand the diversity, evolution and transmission potential of GpSGHV in *Glossina* species. Kariithi et al. [15] reported 23 GpSGHV haplotypes in wild *G. pallidipes* flies, but the virus’ genetic heterogeneity found in that study was low, and without direct correlation to geographical locations. The GpSGHV diversity in other *Glossina* species has yet to be investigated. A high prevalence of both asymptomatic and symptomatic GpSGHV infections in *G. pallidipes* may increase the potential of cross-species exposure and transmission of the virus in mass-rearing facilities where multiple tsetse species are reared. Replication of viruses in a new host species may provide opportunities for the virus to adapt and evolve into novel viral haplotypes that may be more pathogenic, due to accumulated mutations over time. For instance, Grubaugh et al. [16] recently demonstrated that the genetic diversity of West Nile virus (WNV) depended on the mosquito species. The study demonstrated that the southern house mosquito, *Culex quinquefasciatus* Say supported the evolution of WNV variants exhibiting greater fitness when transferred to avian hosts compared to three other *Culex* species. The study concluded that *C. quinquefasciatus* is the main engine that drives WNV evolution. Although the study involved an RNA virus, the same dynamics may apply to evolution of DNA viruses such as GpSGHV, though most likely at much slower evolutionary rates.

In this study, we investigated the GpSGHV genetic diversity and prevalence in seven *Glossina* species obtained from different geographical locations throughout Africa. Based on comparison of the genomes of the two pathogenic strains GpSGHV-Uga and GpSGHV-Eth [16], the three highly diverged GpSGHV genes were selected, as well as two variable number tandem repeat regions (VNTRs) and these were used to construct phylograms and to search for the ancestor of this hytrosavirus. Our data are important for future development of robust strategies to effectively manage GpSGHV infections and sustainably remove SGHV from tsetse mass-rearing facilities.

## Results

### Prevalence of GpSGHV infection in wild Glossina species

GpSGHV prevalence was tested for 3229 flies collected from 29 geographical locations and belonging to seven tsetse species (Table 1). The GpSGHV prevalence of some of the *G. pallidipes* individuals used in the current study was previously reported by Kariithi et al. [15]. In the current study, we extended the number of sampling locations and included other *Glossina* species. GpSGHV prevalence was determined by PCR amplifications of two conserved viral genes the *odv-e66* (SGHV005) and *dnapol* (SGHV079) which were not applied in the genetic diversity analysis. Morphological identification of tsetse species is challenging and sometimes inaccurate. Therefore, on the samples that were positive for GpSGHV, we used PCR generated ITS1 amplicons [17] to assess the taxonomic status of the seven tsetse species (Table 1). In the current study, the ITS1 PCR products were of the expected sizes, which confirmed the status of *G. pallidipes* (920 bp), *G. f. fuscipes* (618 bp), *G. brevipalpis* (778 bp), *G. p. palpalis* (618 bp) *and G. austeni* (633 bp) (Additional file 1; Figure S1A). These sizes were consistent with ITS1 PCR product sizes of known tsetse species reared at the IPCL [17]. The *G. m. morsitans* and *G. swynnertoni* with equal ITS1 sequence lengths (775 bp) were distinguished by the presence of the endosymbiont *Wolbachia* in *G. m. morsitans*, and its absence in *G. swynnertoni* (Additional file 1: Figure S1B). *Wolbachia* integration and prevalence in *G. m. morsitans* was evidenced by the lower (296 bp) and the upper (438 bp) bands on the agarose gel (Additional file 1: Figure S1B**)**. The variations in the *Wolbachia* prevalence agreed with previous studies in field and laboratory tsetse populations [18].

**Table 1:** Details of *Glossina* species sampled in different sites in Africa

Country	Location	Species	Collection date	Latitude	Longitude	Total number	Prevalence (%)
Field collected samples							
Uganda	Tororo	*G. f. fuscipes*	1994	0°41′34.0”N	34°10′52.0″E	17	6 (35.3%)
	Buvuma Island	*G. f. fuscipes* *	1994	0°14′36.7”N	33°16′53.9″E	10	4 (40.0%)
	Kiyindi Island	*G. f. fuscipes*	1994	0°19′20.4”N	32°59′34.2″E	8	2 (25.0%)
	Bagala Island	*G. f. fuscipes*	1994	0°25′15.2”S	32°14′38.1″E	18	5 (27.8%)
Ethiopia	Arba Minch	*G. pallidipes* *	2006	6°07′01.2”N	37°01′60.0″E	431	297 (68.9%)
Kenya	Mwea	*G. pallidipes* *	2007	0°53′15.9”N	37°37′59.7″E	233	17 (7.3%)
	Mwea N. Park	*G. pallidipes*	2008	0°49′23.2”S	37°37′02.3″E	21	1 (4.8%)
	Katotoi	*G. pallidipes*	2007	0°42′42.7”N	34°18′57.1″E	226	0 (0.0%)
	Meru N.Park	*G. pallidipes*	2008	0°05′18.2”N	38°11′23.8″E	95	1 (1.1%)
	Kiria	*G. pallidipes*	2008	0°31′09.8”S	36°37′27.3″E	20	0 (0.0%)
	Koibos Soi	*G. pallidipes* *	2008	0°09′57.9”N	36°06′20.6″E	94	19 (20.2%)
	Mogotio-Emsos	*G. pallidipes* *	2008	0°01′00.4”S	35°57′32.7″E	72	14 (19.4%)
	Ruma N. Park	*G. pallidipes* *	2007	0°38′44.8”S	34°16′31.8″E	176	3 (1.7%)
	Obekai	*G. f. fuscipes*	2007	0°30′52.5”N	34°12′17.6″E	38	0 (0.0%)
	Ikapolok	*G. f. fuscipes*	2007	0°37′44.9”N	34°18′38.0″E	52	0 (0.0%)
Tanzania	Kwekivu	*G. pallidipes* *	2005	5°46′30.5”S	37°23′55.4″E	50	44 (88.0%)
		*G. m. morsitans* *	2005			50	29 (58.0%)
	Kwamume	*G. pallidipes* *	2005	5°41′51.9”S	37°52′01.3″E	33	1 (3.0%)
		*G. m. morsitans*	2005			50	0 (0.0%)
	Ikorongo GR	*G. swynnertoni* *	2015	1°54′58.8”S	34°43′49.8″E	48	23 (47.9%)
	Jozani, Zanzibar	*G. austeni*	1994	6°14′28.4”S	39°24′50.3″E	29	6 (20.7%)
Zambia	Mfuwe	*G. pallidipes* *	2007	13°04′41.2”S	31°47′26.5″E	201	49 (24.1%)
		*G. m. morsitans* *	2007			116	9 (7.8%)
Zimbabwe	Mashumbi	*G. pallidipes* *	2006	15°56′13.8”S	29°27′25.7″E	50	1 (2.0%)
		*G. m. morsitans*	2006			8	0 (0.0%)
	Gokwe	*G. pallidipes* *	2006	17°36′14.5”S	28°27′41.1″E	150	10 (6.7%)
		*G. m. morsitans*	2006			92	23 (25.0%)
	Ruckomechi	*G. pallidipes* *	2006	15°50′55.0”S	29°07′30.0″E	97	30 (30.9%)
		*G. m. morsitans* *	2006			103	21 (20.4%)
	Makuti	*G. pallidipes*	2006	16°17′59.0”S	29°17′59.9″E	96	0 (0.0%)
		*G. m. morsitans* *	2006			99	9 (9.1%)
	Mukondore	*G. m. morsitans*	1995	16°05′22.7”S	29°14′36.0″E	36	18 (50.0%)
	Chiuyi	*G. m. morsitans*	1995	16°6′31.6”S	29°24′33.8″E	36	19 (50.0%)
DRC	Malanga	*G. p. palpalis* *	1995	5°33′26.6”S	14°21′00.1″E	52	4 (7.7%)
South Africa	Zululand	*G. brevipalpis* *	1995	28°01′07.2”S	32°12′52.6″E	33	5 (15.2%)
		*G. austeni* *	1999			53	14 (26.4%)
*Laboratory colonised Glossina species*							
Uganda	Tororo (IPCL)	*G. pallidipes* *	2010	0°41′34.0”N	34°10′52.0″E	48	48 (100.0%)
Kenya	BioRI-KALRO	*G. pallidipes*	2008	1°13′28.0”S	36°38′10.2″E	99	1 (1.0%)
		*G. m. morsitans* *	2008			89	16 (17.9%)
Total						3229	

Samples were collected from different geographical sites in eastern, southern and central African countries for the analysis of GpSGHV prevalence and genetic diversity. Only the samples marked by a star (*) were further analysed for GpSGHV genetic diversity

The GpSGHV prevalence was highest in the *G. pallidipes* colonized at FAO/IAEA Insect Pest Control Laboratory (IPCL) that originated from Tororo, (Uganda; 100%), followed by Kwekivu (Tanzania; 88%), Arba-Minch (Ethiopia; 68.9%), and Ruckomechi (Zimbabwe; 30.9%) (Table 1). The virus was not detected in *G. pallidipes* populations from Kiria and Katotoi in Kenya. The prevalence of the virus in *G. m. morsitans* flies was highest in the field collected samples from Kwekivu (Tanzania; 58%), Chiuyi and Mukondore (Zimbabwe; 52% and 50%, respectively) compared to the laboratory colonised *G. m. morsitans* from BRI-KALRO (17.9%). The virus prevalence varied widely amongst the Ugandan *G. f. fuscipes* specimens (40%, 35.3%, 27% and 25% in Buvuma, Tororo, Bagala Island and Kiyindi Island, respectively), whereas the virus was not detected in the Kenyan *G. f. fuscipes* specimens. The virus prevalence was likewise high (47.9%) in the *G. swynnertoni* specimens from the Ikorongo Game Reserve in Tanzania, but lower in the populations of *G. austeni* (26.4%) and *G. brevipalpis*, (15.2%) from KwaZulu Natal, South Africa, and in specimens of *G. p. palpalis* from Malanga, Democratic Republic of the Congo (7.7%) (Table 1). These results provide evidence that the GpSGHV is present in multiple tsetse species, to varying degrees under laboratory and field conditions.

### Geographical distribution of GpSGHV haplotypes

**Fig. 1:**
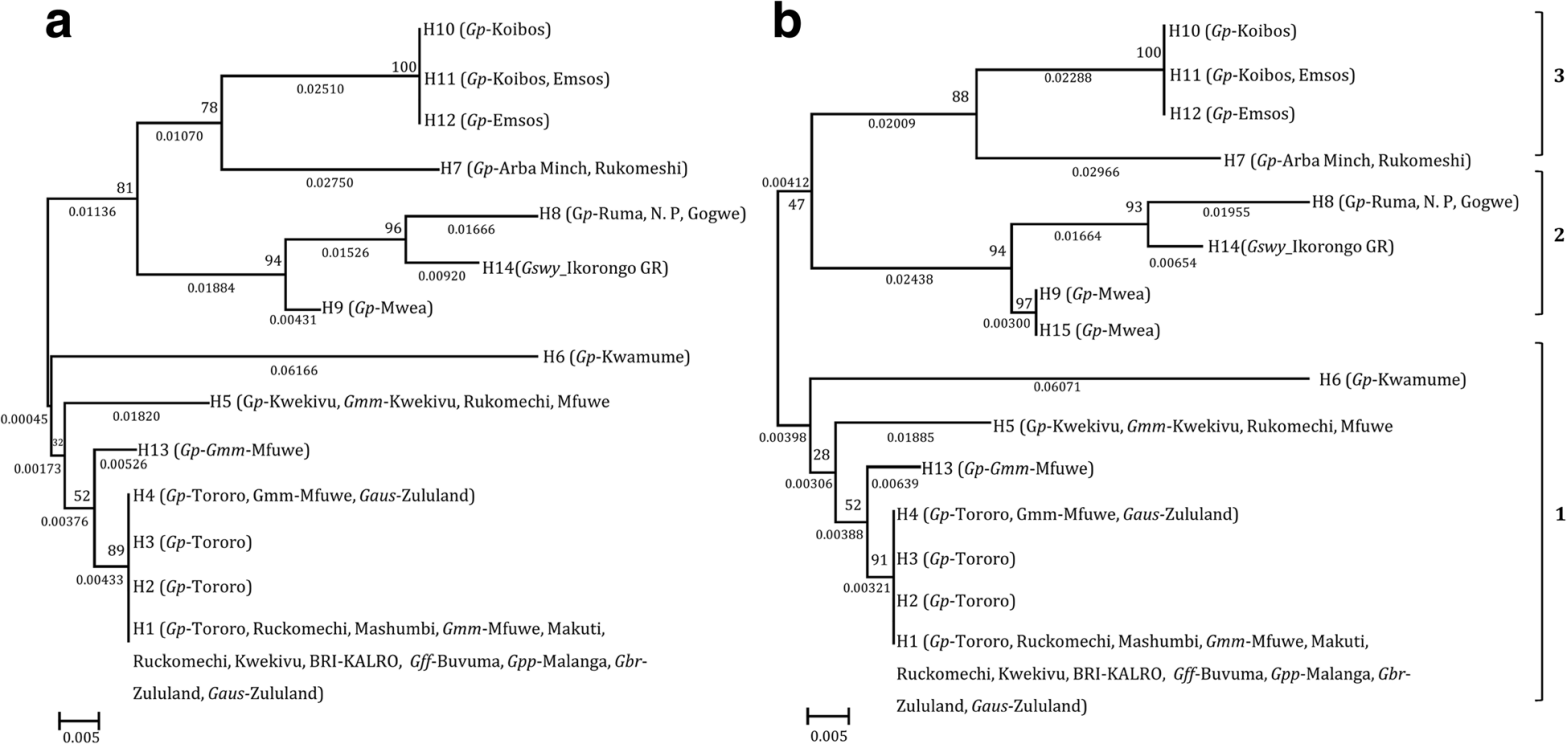
GpSGHV haplotypes in *Glossina* species: Maximum Likelihood (ML) phylogenetic tree for the GpSGHV strains from different geographical locations in Africa using (**a**) VNTR-2 and (**b**) concatenated sequences of VNTR-1, VNTR-2, ORF009, ORF010 and ORF038. ML bootstrap values based on 1000 replicates are shown on the branches. Abbreviations: *Gp (G. pallidipes), Gmm (G. m. morsitans), Gswy (G. swynnertoni), Gff (G. f. fuscipes), Gbr (G. brevipalpis), Gpp (G. p. palpalis), and Gaus (G. austeni)*

**Fig. 2:**
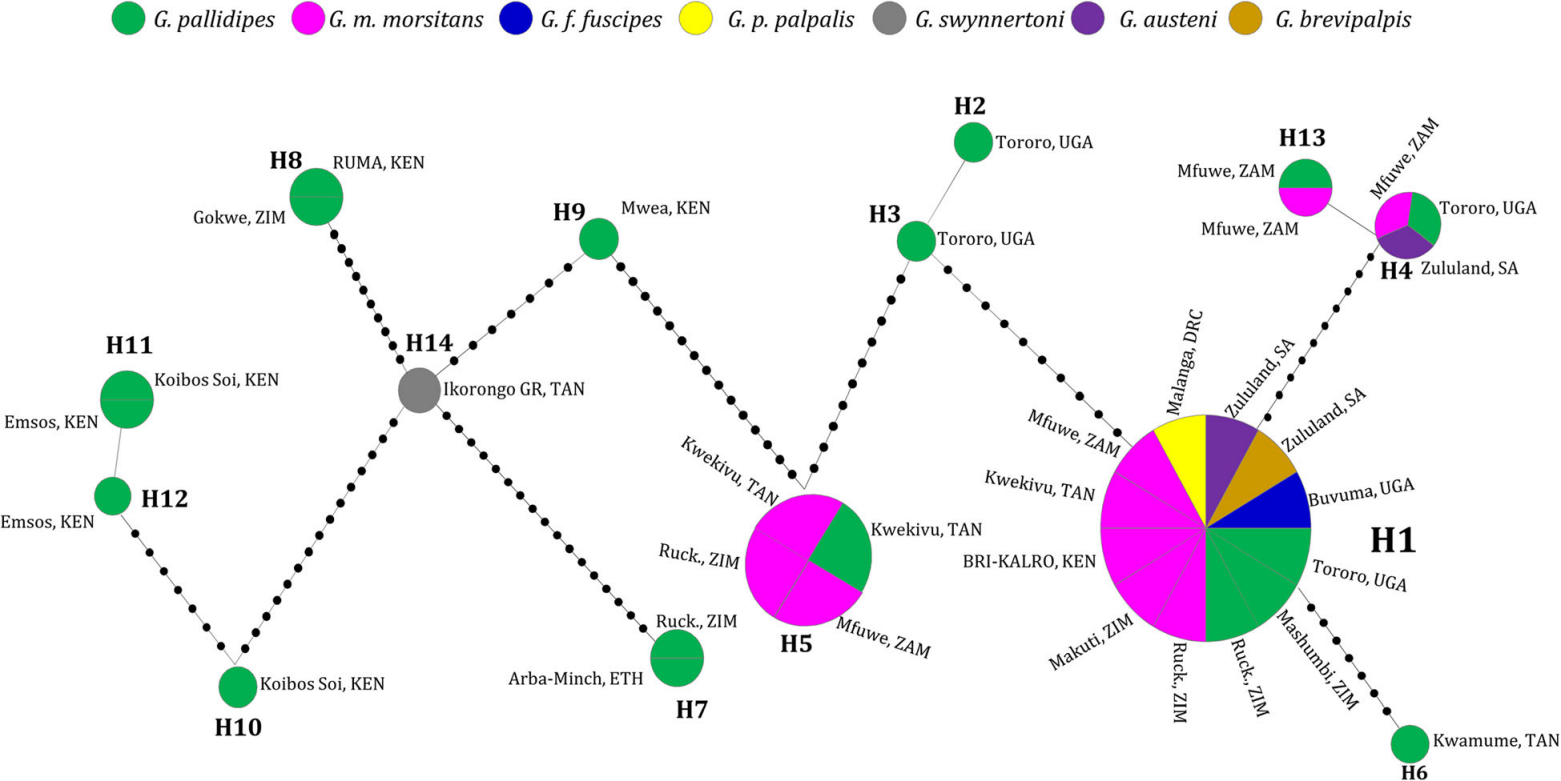
GpSGHV haplotype network in *Glossina* species: The haplotype network generated based on the ML tree generated based on GpSGHV VNTR-2 sequence. The black dots on the lines represent mutations events between the haplotypes. The different colours represent the *Glossina* species. Abbreviations; KEN (Kenya), TAN (Tanzania), ETH (Ethiopia), ZIM (Zimbabwe), ZAM (Zambia), UGA (Uganda), SA (South Africa), DRC (Democratic republic of Congo), Ruck. (Ruckomechi), BRI-KALRO (Biotechnology Research institute- Kenya agricultural and livestock research organization), GR (Game reserve)

The VNTR-2 sequences, which were successfully obtained from all individual GpSGHV positive samples in the seven tsetse species revealed 14 GpSGHV haplotypes (Fig. 1a). Sequence analysis using the alignments of the concatenated sequences of the 3 conserved genes and the two VNTRs revealed 15 GpSGHV haplotypes due to sequence differences found in SGHV009 of *G. pallidipes* samples from Mwea, Kenya (Fig. 1b). However, the relationship between the other 14 haplotypes did not change when the analysis was performed using either the VNTR-2 or concatenated sequences. SGHV009, SGHV010 and SGHV038 and the VNTR-1 of some of the samples could not be sequenced due to failure to amplify the region with the PCR conditions used in the current study. VNTR-2 was the only successfully sequenced region in all the representative samples. The distribution of the identified haplotypes over the *Glossina* species varied depending on the geographical locations (Table 2). Haplotype H1, which corresponded to the GpSGHV-Uga reference [19], was with the exception of *G. swynnertoni*, found in all examined *Glossina* species. This haplotype was found in tsetse populations sampled in 10 different locations in seven of the eight countries (except in Ethiopia) (see Table 2). Seven of the 15 GpSGHV haplotypes (H2, H3, H6, H9, H10, H12 and H15) were restricted to *G. pallidipes* specimens in specific locations. Haplotype H5 was detected in *G. pallidipes* and *G. m. morsitans* from Kwekivu, Tanzania, in *G. m. morsitans* from Ruckomechi, Zimbabwe and from Mfuwe, Zambia (Table 2). Haplotypes H7, H8 and H11 were each detected in *G. pallidipes* specimens from two distinct locations, while H13 was restricted to Zambian *G. pallidipes* and *G. m. morsitans* specimens. Haplotype H14 was restricted to the *G. swynnertoni* samples from the Ikorongo Game Reserve in Tanzania. Notably, in some cases, the same tsetse species (but not the same individual flies) from the same geographical locations harboured more than one haplotype. For example, *G. pallidipes* from Kenya, were infected with H10 and H11 (Koibos-Soi), and H11 and H12 (Emsos) (Fig. 2**)**. Similar observations were made in the haplotypes infecting *G. pallidipes* from IPCL originated from Uganda (H1, H2, H3 and H4).

**Table 2:** Descriptions of the 15 GpSGHV haplotypes (abbreviated by ‘H’) identified in *Glossina* species from different geographical locations

Country	Location	Species	Tested flies	Haplotype (No of tested samples occurring in the haplotype)					Total no. of haplotypes within the *Glossina* species (haplotype name)
				VNTR-1	VNTR-2	SGHV009	SGHV010	SGHV038	
Uganda	Tororo	*G. pallidipes*	8	H1(8)	H1(5), H2(1), H3(1), H4(1)	H1(8)	H1(8)	H1(8)	4(H1, H2, H3, H4)
	Buvuma Island	*G. f. fuscipes*	9	H1(6)	H1(9)	–	–	–	1(H1)
Ethiopia	Arba Minch	*G. pallidipes*	8	H7(8)	H7(8)	H7(8)	H7(8)	H7(8)	1(H7)
Kenya	Mwea	*G. pallidipes*	5	H9(1)	H9(5)	H9(2), H15(1)	H9(3)	H9(3)	2(H9, H15)
	Koibos Soi	*G. pallidipes*	8	–	H10(7), H11(1)	H11(4)	H11(4)	H11(4)	2(H10, H11)
	Emsos	*G. pallidipes*	8	–	H11(7), H12(1)	H11(2)	H11(2)	H11(2)	2(H11, H12)
	BioRI-KALRO	*G. m. morsitans*	16	H1(12)	H1(16)	–	–	–	1(H1)
	Ruma N Park	*G. pallidipes*	1	–	H8(1)	–	H8(1)	H8(1)	1(H8)
Tanzania	Kwamume	*G. pallidipes*	1	H6(1)	H6(1)	–	–	–	1(H6)
	Kwekivu	*G. pallidipes*	8	H5(4)	H5(8)	H5(6)	H5(6)	H5(6)	1(H5)
		*G. m. morsitans*	17	H1(1)	H1(6), H5(11)	H1(4)	H1(4)	H1(4)	2(H1, H5)
	Ikorongo GR	*G. swynnertoni*	3	–	H14(3)	H14(3)	H14(3)	H14(3)	1(H14)
Zambia	Mfuwe	*G. pallidipes*	4	–	H13(4)	H1(4)	H1(4)	H1(4)	2(H1, H13)
		*G. m. morsitans*	9	H1(7)	H13(6), H1(1), H4(1), H5(1)	–	–	–	4(H1, H4, H5, H13)
Zimbabwe	Mashumbi	*G. pallidipes*	1	–	H1(1)	–	–	–	1(H1)
	Gokwe	*G. pallidipes*	1	–	H8(1)	–	–	–	1(H8)
	Ruckomechi	*G. pallidipes*	8	H1(3)	H1(7), H7(1)	H1(3)	H1(3)	H1(3)	2(H1, H7)
		*G. m. morsitans*	14	H1(12)	H1(13), H5(1)	H1(3)	H1(3)	H1(3)	2(H1, H5)
	Makuti	*G. m. morsitans*	8	–	H1(8)	H1(1)	H1(1)	H1(1)	1(H1)
DRC	Malanga	*G. p. palpalis*	4	H1(3)	H1(4)	–	–	–	1(H1)
South Africa	Zululand	*G. brevipalpis*	5	H1(4)	H1(5)	–	–	–	1(H1)
		*G. austeni*	6	H1(2)	H1(5), H4(1)	–	–	–	2(H1, H4)

The numbers between the brackets in columns 4 to 8 indicate the number of samples presenting a particular haplotype. Column 10 shows the total number of haplotypes found in each tsetse species in different locations, and the abbreviated names of the GpSGHV haplotypes in this column are indicated in the brackets

### Single nucleotide polymorphisms (SNPs) and nonsynonymous mutations among GpSGHV haplotypes

After analysing the GpSGHV haplotype distribution amongst the *Glossina* species from the different geographical locations (Table 3), we determined synonymous and nonsynonymous single nucleotide polymorphisms (SNPs) in the amplified nucleotide sequences. The analysis revealed a high rate of SNPs, and a few deletions and insertions (Table 3). Haplotype H1, which is similar to the reference GpSGHV-Uga, remained without mutations within the haplotype. Most of the deletions and insertions were found in the VNTRs, with the VNTR-2 presenting most of the inter- and intra-haplotype variations. The VNTRs mutations observed were mostly patterns in repeat polymorphisms, i.e. additions or deletions of repeat units, rather than single nucleotide mutations (Additional file 2: Figure S2B). VNTR-2 of H7, which infected the Ethiopian (Arba Minch) and Zimbabwean (Ruckomechi) *G. pallidipes* populations, harboured the most intra-haplotype variation (i.e. 24 SNPs, and 126 bp and 20 bp insertions and deletions, respectively) (Table 3). Compared to the GpSGHV-Uga reference sequence, most of the nonsynonymous mutations of 19 of 178, 12 of 475 and 10 of 489 amino acids in SGHV009, SGHV010 and SGHV038, respectively, were found in the H7 infecting the Ethiopian (Arba Minch) and Zimbabwean (Ruckomechi) *G. pallidipes* populations (Table 3). Further, H10, H11 and H12 infecting *G. pallidipes* populations from Kenya (Koibos-Soi and Emsos) also presented high mutational variations within the haplotype, with five of 178, 12 of 475 and nine of 489 amino acids of nonsynonymous mutations in SGHV009, SGHV010 and SGHV038, respectively. The greatest number of nonsynonymous mutations was found in the haplotypes infecting *G. pallidipes* populations, followed by those infecting the *G. m. morsitans* populations (see Table 3).

**Table 3:** Analysis of PCR product lengths in nucleotides, single nucleotide polymorphisms (SNPs), insertion and deletions detected in the GpSGHV haplotypes in the *Glossina* species

Haplotype	Species	Location	PCR Length: (SNPs, insertion, deletion) (bp)					No. of nonsynonymous mutations (aa)
			VNTR-1	VNTR-2	SGHV009	SGHV010	SGHV038	(SGHV009, SGHV010, SGHV038)
GpSGHV-Uga	*Reference*	NCBI	370	437	535	1425	1461	Reference
H1	*G. pallidipes*	Tororo	370: (0,0,0)	437: (0,0,0)	535: (0,0,0)	1425: (0,0,0)	1461: (0,0,0)	(0,0,0)
	*G. m. morsitans*	Ruckomechi						
	*G. m. morsitans*	Mfuwe						
	*G. m. morsitans*	Makuti						
	*G. pallidipes*	Ruckomechi						
	*G. pallidipes*	Mashumbi						
	*G. m. morsitans*	BioRI-KALRO						
	*G. m. morsitans*	Kwekivu						
	*G. f. fuscipes*	Buvuma Island						
	*G. p. palpalis*	Malanga						
	*G. brevipalpis*	Zululand						
	*G. austeni*	Zululand						
H2	*G. pallidipes*	Tororo	370: (0,0,0)	500: (2,63,0)	535: (0,0,0)	1425: (0,0,0)	1461: (0,0,0)	(0,0,0)
H3	*G. pallidipes*	Tororo	370: (0,0,0)	500: (0,63,0)	535: (0,0,0)	1425: (0,0,0)	1461: (0,0,0)	(0,0,0)
H4	*G. pallidipes*	Tororo	370: (0,0,0)	374: (0,0,63)	535: (0,0,0)	1425: (0,0,0)	1461: (0,0,0)	(0.0,0)
	*G. m. morsitans*	Mfuwe						
	*G. austeni*	Zululand						
H5	*G. pallidipes*	Kwekivu	416: (12,66,20)	542: (15,105,0)	538: (1,3,0)	1425: (4,0,0)	1467: (12,6,0)	(1,1,4)
	*G. m. morsitans*	Kwekivu						
	*G. m. morsitans*	Ruckomechi						
	*G. m. morsitans*	Mfuwe						
H6	*G. pallidipes*	Kwamume	415: (13,65,20)	458: (14,21,1)	?	?	?	?
H7	*G. pallidipes*	Arba Minch	272: (8,1,99)	542: (24,126,20)	571: (14,36,0)	1425: (16,0,0)	1458: (26,0,3)	(19,12,10)
	*G. pallidipes*	Ruckomechi						
H8	*G. pallidipes*	Gokwe	?	483: (47,46,0)	?	1425: (14,0,0)	1460: (14,0,0)	(?,9,2)
	*G. pallidipes*	Ruma N. Park						
H9	*G. pallidipes*	Mwea	431: (11,61,0)	521: (30,84,0)	523: (7,0,12)	1425: (11,0,0)	1460: (20,0,1)	(3,7,5)
H10	*G. pallidipes*	Koibos Soi	?	458: (40,21,0)	529: (6,3,9)	1425: (15,0,0)	1461: (21,0,0)	(5,12,9)
H11	*G. pallidipes*	Koibos Soi	?	416: (36,0,21)	529: (6,3,9)	1425: (15,0,0)	1461: (21,0,0)	(5,12,9)
	*G. pallidipes*	Emsos						
H12	*G. pallidipes*	Emsos	?	416: (35,0,63)	529: (6,3,9)	1425: (15,0,0)	1461: (21,0,0)	(5,12,9)
H13	*G. pallidipes*	Mfuwe	?	374: (3,0,63)	535: (0,0,0)	1425: (0,0,0)	1461: (0,0,0)	(0,0,0)
	*G. m. morsitans*	Mfuwe						
H14	*G. swynnertoni*	Ikorongo GR	?	503: (36,67,0)	532: (3,0,3)	1425: (14,0,0)	1467: (16,6,0)	(1,9,6)
H15	*G. pallidipes*	Mwea	431: (11,61,0)	521: (30,84,0)	532: (3,3,6)	1425: (11,0,0)	1460: (20,0,1)	(2,7,5)

The polymorphisms were based on the partial sequences of GpSGHV ORFs SGHV009, SGHV010, SGHV038, VNTR-1 and VNTR-2. The 3 numbers in brackets for each haplotype in column four to column eight refer to the number of SNPs, insertions and deletions respectively, relative to the GpSGHV-Uga reference sequences. The samples marked with a question mark (?) were not successfully sequenced. The nonsynonymous mutations of SGHV009, SGHV010, SGHV038 found in each sample are also shown in the last column

### Phylogenetic analysis of GpSGHV haplotypes

Analysis of the phylogenetic relationships of the various GpSGHV haplotypes revealed three distinct clades consisting of haplotypes from different locations (Fig. 1). The largest clade (clade 1) consisted of haplotypes H1, H2, H3, H4, H5, H6 and H13 infecting six out of seven *Glossina* species derived from most of the geographical locations. Clade 2 consisted of H8, H9, H14 and H15, which infected *G. pallidipes* from Mwea, Ruma (Kenya), Gokwe (Zimbabwe) and *G. swynnertoni* from the Ikorongo Game reserve (Tanzania). The third clade included H7, H10, H11 and H12 infecting *G. pallidipes* samples from Koibos-Soi, Emsos (Kenya), Arba Minch (Ethiopia) and Ruckomechi (Zimbabwe) (Fig. 1). The clustering of the Kenyan haplotypes (H10, H11 and H12, infecting *G. pallidipes*) in clade 3, which was supported by 100% bootstrap values, appeared to be closely related to the Ethiopian H7. H7 is presumably the GpSGHV-Eth strain, whose genome has been fully sequenced and which might be more pathogenic than the reference GpSGHV-Uga strain (H1) [11].

### Potential ancestry of GpSGHV haplotypes

To gain insights into the evolutionary history of GpSGHV, which can be seen as a series of mutation events leading to the various haplotypes, we analysed the genealogies (or gene trees) [20] using both VNTR-2 alone (Fig. 2**)** and the concatenated gene sequences of the above-mentioned three genes and the two VNTRs (Additional file 3: Figure S3). The haplotype genealogies did not differ when analysed using either the VNTR-2 or the concatenated sequences. In order to be able to include also H15, which is similar to H9 in VTNR-2 sequence and only differ at SGHV009 sequence, the concatenated sequences were used for the analysis. The topology of the star-like genealogies revealed three potential ancestral origins of the various GpSGHV haplotypes (Fig. 2), which were largely in agreement with the clustering observed in the phylogeny (Figs. 1 and 2). Due to its large host range and wide geographical representations, we presumed H1 to be the ancestral origin of all the 15 haplotypes (i.e. shared parental DNA sequences). This would be in line with the wide distribution of H1 described above (in six of the seven *Glossina* species, originating from 10 of geographical locations (Fig. 2). Further, H6 (infecting *G. pallidipes* only), H4 (infecting *G. pallidipes*, *G. m. morsitans* and *G. austeni*), and H13 (infecting *G. pallidipes* and *G. m. morsitans*) potentially trace back to H1 by eight and 11 mutation events, respectively. H5 was the second potential ancestral haplotype origin. Notably, based on the analysed genes in this study, H2 and H3 (infecting the IPCL *G. pallidipes* populations) presumably coalesced from H1 and H5 by 11 and 10 mutation events, respectively (Fig. 2; see also Table 3). On the other hand, H14, which infects the *G. swynnertoni* from the Ikorongo Game reserve in Tanzania, can be interpreted to be the ancestral origin of H7, H8, H9, and H10. Additionally, H9 and H15 (Additional file 3: Figure S3) potentially coalesced from H5 and H14 by 7–8 and 15–16 mutation events, respectively. Finally, H10, H11 and H12 from Kenya showed the highest divergence from H1, which as mentioned above, corresponded to the reference GpSGHV-Uga.

## Discussion

This study provides the first evidence that all seven *Glossina* species examined harboured GpSGHV. The ability of GpSGHV to infect multiple *Glossina* species is important because the virus could hamper future SIT efforts as part of AW-IPM programmes against specific *Glossina* species in various sub-Saharan African countries. This finding is especially relevant for tsetse mass production facilities where multiple tsetse species are reared and often receive their blood meals using the same membranes in successive feeding cycles [21]. This feeding regime increases risks of virus transmission within and between the species. This cross-species virus transmission could result in viral amplifications and SGH outbreaks in laboratory colonies or mass rearing facilities (due to horizontal transmission of infectious virus particles via saliva during the in vitro membrane feeding [12]). Additionally, the cross-species virus transmission, could result in the generation within the original and new hosts of virus variants capable of efficient spreading amongst multiple host species [22].

The finding that GpSGHV prevalence was highest in *G. pallidipes* irrespective of the geographical locations (e.g. 100% in IPLC colony and 88% in Tanzania field samples) implies that the virus is present at high frequency in this species. This finding supports the view that this virus most probably has a recent relationship with *G. pallidipes* compared to other tsetse species analysed in this study, perhaps due to virus-host interactions that influence genetic drift and selection as reported for arbovirus infection in different mosquito species [16]. Next to *G. pallidipes*, the virus prevalence was high amongst populations of *G. m. morsitans* (e.g. 58% in Tanzania and 20% in Zimbabwe), and *G. f. fuscipes* (25–40% in Uganda). It should be noted that SGH symptoms were first reported in the 1930’s amongst *G. pallidipes* populations in the Umfolozi Game Reserve, KwaZulu Natal, South Africa [23], and later in the early 1970’s the causative virus was observed in *G. morsitans* and *G. f. fuscipes* in Tanzania and Uganda, respectively [24, 25] Later, these virus particles were associated with SGH in *G. pallidipes* [26]. Decades later (in 1993), the virus was reported in *G. brevipalpis* populations in Kenya [27]. Overall, based on the chronological history since the initial discovery of SGH in *Glossina* [26], unlike others tsetse species, *G. pallidipes* is evidently the most common *Glossina* species to which GpSGHV has not yet evolutionarily adapted to [12]. It is yet to be determined why the virus is more pathogenic to *G. pallidipes* as compared with other *Glossina* species. It is obvious that a well-established evolutionary relationship between the virus and the host will result in a stable status, where the virus can be present in the host without affecting the host’s general fitness or causing disease symptoms. What is known is that pathogens, including viruses, can specifically modulate their host-environment infections to favour their transmission [28].

The largest number of nonsynonymous mutations (in all the three genes) was found within the GpSGHV haplotypes infecting *G. pallidipes* populations from Ethiopia, Zimbabwe (H7), and Kenya (H10, H11 and H12). The nonsynonymous mutations provide a preview of the evolutionary path that can shape the genetic structure of viral haplotypes [29]. Of the two GpSGHV strains whose genomes have been fully sequenced, the Ethiopian strain (GpSGHV-Eth) had a higher (> 85%) SGH prevalence in *G. pallidipes* originating from Ethiopia, than the Ugandan strain (GpSGHV-Uga) infecting *G. pallidipes* from Uganda (10% SGH prevalence), despite the two colonies being maintained in the same insectary conditions in IPCL [11, 21, 30]. Whereas higher SGH incidences may not necessarily reflect higher pathogenicity, there are indications that this may be the case. It could be as well that the *G. pallidipes* flies from Ethiopia are less tolerant to the virus infection compared to the long domesticated *G. pallidipes* colony in IPCL [21]. In the current study, VNTR-2 revealed the highest rate of deletions and insertions of repeat units. VNTRs are amongst the most discriminating of the genotyping methods, and have been used in pedigree analysis of disease-causing pathogens due to their roles in rapid genome evolution and adaptations [31]. Although VNTRs usually generate neutral genetic variations [32], some VNTRs can alter critical biological functions. For instance, if localized near or within gene promoter regions, VNTRs may affect transcription of downstream genes by affecting the number of transcription factor binding sites, or inducing changes in spacing between critical promoter elements [33]. In addition, it has been reported that polymorphism in VNTR loci contributes to genome evolution [31]. In this study, the sequence analysis using either the VNTR-2 or the concatenate sequences of the three genes and two VNTRs revealed the same haplotypes. This indicates that VNTR-2 can be a suitable tool/ microsatellite to discriminate GpSGHV haplotypes. Of the three genes used in this study, SGHV010 and SGHV038, which code for putative desmoplakin-like protein and maltodextrin glycosyltransferase, respectively, are both virion tegument proteins [11, 34]. For some DNA viruses (e.g. herpesviruses), tegument proteins have been described that are essential for virus replication [35]. The third gene (SGHV009) is known to be homologues to viral regulatory proteins. Although the mutations in the three ORFs and the two VNTRs represent a small subset of the GpSGHV genome to make robust conclusions, we hypothesize that these mutations might affect the pathogenesis of GpSGHV-Eth as compared to the GpSGHV-Uga. It would be interesting to sequence the genomes of the GpSGHV strains circulating amongst the Zimbabwean and Kenyan *G. pallidipes* populations. This will help determine their genetic differences, compared to already sequenced GpSGHV strains. The nonsynonymous mutations found in these haplotypes need further investigations to elucidate their impacts on the virus pathobiology.

The central genealogy hubs (based on the haplotype network) occupied by haplotypes H1 (infecting six of the seven analysed *Glossina* species), H5 (infecting three *Glossina* species), and H14 (infecting only *G. swynnertoni*) provided insight into the potential ancestral origins and evolution of GpSGHV in *Glossina*. In addition, haplotype H1 is presumed to be the best potential ancestor of the virus among the three haplotypes due to its wide host-range and geographical representation. Hence, it was difficult and beyond the scope of this paper to trace the origin and evolution of the virus through the species host or the geographical locations. However, an accurate assessment of the origin and evolution of large dsDNA viruses needs to be based on whole genomes, including primary genomic sequence comparisons, genome organizations and gene content [36]. We have recently shown that the two GpSGHV strains (GpSGHV-Uga and GpSGHV-Eth), although similar in nucleotide sequence (98.1%), differ in their genomes in terms of the numbers of ORFs (with insertions and deletions of entire ORFs), and SNPs within the genes [11]. We selected the three genes and the two VNTRs for our analyses because they exhibited the most significant differences between the two virus strains. Our hypothesis that H1 and H5 are potentially of ancestral origin is supported by previous findings by Kariithi et al. [15], who found the same haplotypes to occupy similar positions in the genealogical network. However, H5 from the previous study was found in *G. pallidipes* from Kenya, while in the current study H5 infected both *G. m. morsitans* and *G. pallidipes* from Tanzania, and *G. m. morsitans* from Zimbabwe and Zambia. In the current study, we have identified H14 (infecting *G. swynnertoni*) as an additional possible ancestral GpSGHV origin. Further studies are necessary to characterize the haplotypes found in Kenya, which we found to be phylogenetically related to the GpSGHV-Eth, as well as to H14. Several authors from Kenya reported the occurrence of SGH symptoms in wild-caught *Glossina* species [See the summary in Table 1 from Kariithi et al.,] [12]. Since the occurrence of SGH symptoms is an exception [37], especially in wild tsetse populations, one could conclude that the GpSGHV strains circulating amongst the various tsetse populations in Kenya could be as pathogenic as the GpSGHV-Eth. Although we detected multiple GpSGHV haplotypes infecting the same tsetse species from the same geographical location, the occurrence of multiple GpSGHV haplotypes in single individuals was not tested. However, this phenomenon in the *Glossina*-GpSGHV system cannot be ruled out since multiple virus haplotypes in the same individual has been reported in other systems such as *Drosophila*-Drosophila C virus system [38]. Infection by multiple genotypes of nucleopolyhedrovirus has also been reported in *Spodoptera frugiperda* which contributed to the diversity of the virus [39].

These findings suggest that compared to other tsetse species included in this study, *G. pallidipes* might be the most recent host for GpSGHV. The large number of haplotypes observed in *G. pallidipes* suggests that the virus is still in the process of adapting to the host, explaining also why SGH symptoms were first observed in this species. This indicates that the original GpSGHV host species could be any other tsetse species that has yet to present overt SGH symptoms, or is even infected at levels that are too low to be detected by conventional PCR. We hypothesize that the virus might on rare occasions be transmitted horizontally between individuals and species when tsetse flies acquire a blood meal on the same animal in the field. In this scenario, infectious GpSGHV particles can pass from infected to uninfected flies via salivary secretions as up to 10^6^ viral copies are secreted by an infected symptomatic fly in a 10–15 min blood meal feeding event during membrane feeding in the laboratory [21, 30, 40]. These secreted virus particles can be infectious as evidenced by the reduction in virus copy numbers in flies fed with new blood at every feed, compared to flies fed under the normal feeding regime of feeding several sets of cages on the same tray of blood. [21, 30, 40]. In the field, tsetse flies aggregate on specific parts of the host to feed [41, 42] and produce pharmacologically active saliva components that are deposited by the flies at the feeding site to interfere with host responses such as vasoconstriction and thrombocyte aggregation. This helps create a blood pool at the bite site and maintain blood fluidity as well as reducing the blood diffusion rate [43]. This may reduce the dilution by the host animal at the bite site of any infectious viral particles released via the saliva of infected flies and hence increase the chances of horizontal virus transfer to the flies feeding in the bite site proximity. This hypothesis has been discussed previously [6], but needs experimental validation. Virus transmission through a shared food source has been demonstrated in the closest relative of GpSGHV, the MdSGHV that infects houseflies, whereby healthy flies became infected after they were fed on food contaminated by infected flies [44, 45]. Similar modes of virus transmission have been reported in other insect viruses such as Israeli acute paralysis virus (IAPV) in bumblebees [46].

## Conclusions

We have demonstrated that the GpSGHV diversity is higher in *G. pallidipes* compared to other *Glossina* species. However, the high virus diversity in *G. pallidipes* from the current study differed with the results obtained in the previous study by Kariithi et al., [12], which was based on conserved virus genes (*p74*, *pif-1*, *pif-2*, *pif-*3, and *dnapol*). In addition, our results appear to support the concept that GpSGHV has over evolutionary times reached a stable but dynamic equilibrium with *Glossina* species other than *G. pallidipes*. In *G. pallidipes* the virus seems to be undergoing co-adaptation, thus accounting for the higher prevalence and diversity. This concept is also supported by the fact that it is only in *G. pallidipes*, that under certain laboratory settings, support symptomatic SGHV infections. In the natural tsetse populations, SGH symptoms are rarely observed. Taken together, these two studies present VNTR-2 as a potential candidate to distinguish virus haplotypes since it was successfully sequenced in all the analysed individuals. This is as opposed to the use of the concatenated sequences that had missing sequences of VNTR-1, SGHV009, SGHV010 and SGHV038 in some individuals due to unsuccessful attempts to amplify these candidate genes.

The finding that GpSGHV infects all *Glossina* species included in the current and previous studies underscores the importance of taking appropriate measures to ensure that field-derived biological material to establish new tsetse colonies for mass rearing is free of GpSGHV infections. A positive note on virus management in tsetse mass rearing is that it is highly likely that the key GpSGHV genes critical for the virus infections and transmission are conserved over haplotypes, implying that a common strategy can be used to mitigate virus infections in multiple tsetse species. This approach is supported by successful control of the GpSGHV using antiviral drugs, which target the viral *dnapol* gene [21, 47, 48]. With the potential of the evolving viral genotypes with enhanced infection and transmission dynamics in sinsect mass production facilities, the data presented herein are essential for future development of robust strategies against new GpSGHV strains. It is therefore recommended that different tsetse species should be reared in separate insectaries (or under appropriate conditions) to avoid horizontal transmission of GpSGHV from one species to another during membrane feeding.

## Methods

### Sample collection

*Glossina* flies were collected between 1994 and 1995 and between 2005 and 2015 from 29 geographical locations in eastern, southern and central African countries. The flies were collected as described by Kariithi et al., [15]. Flies from seven species were analysed in this study, i.e. *G. pallidipes*, *G. morsitans morsitans*, *G. swynnertoni, G. fuscipes fuscipes*, *G. brevipalpis*, *G. palpalis palpalis* and *G. austeni* **(**Table 1**)**. The collected samples were preserved in absolute ethanol, or propylene glycol, shipped to the IPCL of the Joint FAO/IAEA Division of Nuclear Techniques in Food and Agriculture, Seibersdorf, Austria, and stored at -20 °C until further analysis.

### DNA extraction, polymerase chain reaction (PCR) and gel electrophoresis

Total DNA was extracted from whole fly bodies of 3229 individuals divided over the varies species mentioned above using the DNeasy Tissue Kit (QIAGEN Inc., Valencia, CA) following the manufacturer’s instructions. PCR amplifications were performed as previously described [15]. Briefly, final reaction volumes of 25 μl were used containing 12.5 μl of *Taq* PCR Master Mix (*Taq* PCR Master Mix Kit, QIAGEN Inc.), ~ 50 ng of the isolated DNA template, 1 μl of forward and reverse primers to a final concentration of 0.2 mM per primer and 10 μl of RNAse-free water. PCR products were analysed by 1.5% agarose gel electrophoresis according to standard protocols.

### Verification of the taxonomic status of tsetse species

Taxonomic status of the *Glossina* flies, determined initially on visual identification in the field, were analysed (8/species) using the optimized molecular markers described by Augustinos et al. [17]. The markers consisted of PCR-based sequencing of the non-coding internally transcribed spacer-1 (ITS1) of the ribosomal DNA (rDNA), and on *Wolbachia* diagnosis. The ITS1 sequence provides differences in the PCR product lengths produced with different tsetse species. Diagnosis of *Wolbachia* infection was applied to further verify the tsetse species, i.e. PCR-detection for the presence or absence of this endosymbiont [49]. Laboratory tsetse flies of known taxonomic status (i.e. obtained from the IPCL) were used as positive controls during the species identification by the above-mentioned molecular markers.

### Determination of GpSGHV prevalence

To determine GpSGHV prevalence in the randomly collected tsetse samples, PCRs were performed to amplify a partial sequence of two conserved viral genes, *odv-e66* (SGHV005) and *dnapol* (SGHV079) using sets of primers as described by Abd-Alla et al. [5]. Samples were considered virus-infected if the expected PCR products of at least one of the two viral genes were detected **(**Table 4**)**. The *Glossina* species microsatellite GpCAG133 was used to control the quality of the extracted DNA and the PCR amplifications.

**Table 4:** List of primers used in the study

A. Primers for GpSGHV screening								
Target gene				Forward and Reverse primer sequences (5′ to 3′)	Amplicon size (bp)			Reference
GpCAG133				GpCAG133F ATTTTTGCGTCAACGTGA	180–220			
				GpCAG133R ATGAGGATGTTGTCCAGTTT				
GpSGHV (ODV-e66)				GpSGHV-2F- CTTGTCAGCGCCACGTACAT	401			[5]
				GpSGHV-2R- GCATTCACAGCATCCCAATTTT				
GpSGHV (DNA*-pol*)				83F_GTACATATTCGAATGTATTTGCCGTTGCTC	320			
				82R_CGGGAGGAGTTGTAATACCCTGTATCAAAG				
B. Primers for *Glossina* species identification								
Nuclear marker (ITS1)				*Glossina*ITS1_for- GTGATCCACCGCTTAGAGTGA	Variable (Species specific)			[56]
				*Glossina*ITS1_rev- GCAAAAGTTGACCGAACTTGA				
*Wolbachia* infection status (16S rRNA)				WspecF -YATACCTATTCGAAGGGATAG	438 + (296)			[57]
				WspecR- AGCTTCGAGTGAAACCAATTC				
C. Primers for GpSGHV genetic diversity								
GpSGHV-Uga		GpSGHV-Eth		Forward and Reverse primer sequences	Amplicon size (Eth/Ug) bp	SNPS (bp)	Deletions (bp)	Insertions (bp)
ORF	Position	ORF	Position					
SGHV009	8631> 10,868	SGHVEth008	8634> 10,868	8F_TTTCCTCCAATTCTTCTCTGGCAGC	1433/1436	22	38	35
				8R_CCACGTCAATGTTGCCTTTCAAATC				
SGHV010	14,205 < 10,894	SGHVEth009	14,184< 10,894	11F_GCCGTTTCTTTTCTAATTTCTTCATCTTCGGG	1631/1655	35	24	0
				11R_GCTCAATAGTTTAAAGCACTGTAACCGCGTTGATT				
SGHV038	44,374 < 40,853	SGHVEth039	44,292< 40,702	32F_ACGCTGAACTAAATTATCGTCATCTACACG	1623/1626	28	3	0
				32R_GCACCAATTGAACATGGATTCCGTTAT				
VNTR-1	22,536 > 22,814	VNTR-1	22,617> 22,830	18F_TGGCCCAGCCCTAAATATCTTAATAGCG	511/682	22	170	0
				17R_CAAAGCTGGGCCATATATTGGGTAGAAATT				
VNTR-2	73,504 > 73,727	VNTR-2	73,389> 73,702	R2-Nested3F_GATACGTCTCACTCATACAATC	812/707	42	0	105
				R2-Nested3R_CATATTACCGACAGAGGGCGTTCAC				

The expected PCR product and the variations (SNPs, deletions or insertion) between the Ugandan and Ethiopian strains are indicated

### PCR product purification and sequencing

To determine GpSGHV genetic variation in the virus positive flies (marked with asterisks (*) in Table 1), three putative open reading frames (ORFs) and two VNTRs were selected, based on the differences in the genomes of the virus from Uganda (GpSGHV-Uga; Accession Number: EF568108) and from Ethiopia (GpSGHV-Eth; Accession Number: KU050077) [11]. The selected ORFs were; SGHV009, SGHV010, SGHV038, and the GpSGHV VNTR-1 and VNTR-2 (corresponding to R1 and R2) loci as described in Abd-Alla et al. [19]. PCRs were performed as described above with primers shown in Table 4. The PCR amplification conditions for ORFs SGHV009, SGHV010 and SGHV038 were, 5 min at 95 °C, 35 cycles of 94 °C for 45 s, 60 °C for 45 s and 72 °C for 2 min, then 72 °C for 10 min. PCR cycling conditions for the VNTR-1 and VNTR-2 were, 5 min at 95 °C, 35 cycles of 94 °C for 45 s, 45 °C for 45 s and 72 °C for 1 min, followed by 72 °C for 10 min. PCR products were subsequently purified using the QIAquick PCR purification Kit (Qiagen, Valencia, CA), and sequenced from both ends by the Sanger method (Eurofins Genomics, Ebersberg, Germany) using their respective primer sets.

### Phylogenetic analysis

DNA sequence reads from the sequenced PCR products were assembled and aligned using the SeqMan Pro (Lasergene 14, DNASTAR, Inc.). Only sequences with good quality reads in the chromatograms were further analysed. Single nucleotide polymorphisms (SNPs), deletions or insertions of sequences were determined based on the GpSGHV-Uga genome as the reference. The Open Reading Frame Finder platform (https://www.ncbi.nlm.nih.gov/orffinder/) was used to identify the ORF of SGHV009, SGHV010 and SGHV038 viral genes for all the samples. The nucleotide sequences were translated using the BioEdit program [50] to identify the synonymous and non-synonymous mutations. Both nucleotide and the translated amino acid sequences of individual viral genes were used to determine phylogenetic relationships amongst the GpSGHV haplotypes infecting the seven *Glossina* species. Here, we define a haplotype as a population of closely related genetic variants resulting from mutations events. The sequences were aligned and trimmed using ClustalW on MEGA6 using default settings [51]. Concatenated sequences of the three genes and the two VNTRs were used for phylogenetic analyses using Maximum-Likelihood (ML) based on the General Time Reversible (TR) model with gamma distributed rates [52] with 1000 bootstrap replications. Samples that could not be sequenced in all the selected genes and VNTRs were marked with ‘?’ to indicate missing sequence in the concatenated sequence alignment. It should be noted that attempts to PCR-amplify these samples using flanking primer sets also failed. To determine the number of GpSGHV haplotypes present amongst the *Glossina* populations, samples presenting the same sequence were categorised as a single haplotype.

### Estimation of gene genealogies

The Arlequin software version 3.5 [53] was used to compare the genetic differences of the haplotypes and their relationships (mutation events between the haplotypes). The Arlequin program produced connection lengths which were equal to the number of mutational differences (single nucleotide polymorphisms and deletions/insertions) between two haplotypes, here named as mutation events. The deletions or insertions that occurred as a block of repeat unit in the VNTRs sequences were interpreted as one connection length (mutation event). The Arlequin output files were used to visualize the haplotype network on the HapStar program version 0.7 [54]. Hapstar uses a spring model algorithm by automatic repulsion of disconnected haplotype branch nodes and the connected ones to an optimal format. The haplotype network was then exported as a scalable vector graphics (SVG) and loaded into Inkscape graphics editor software v 0.92.1 [55] for additional text, colours and patterns.

## Additional files

Additional file 1: **Figure S1.** Analysis of PCR-amplification products on 1.5% agarose gels: Panels A and B show the different band sizes of ITS1 sequences and *Wolbachia* diagnosis for different *Glossina* species, respectively. Four samples were analysed to represent each species from different geographical regions. The positive controls were *Glossina* species from the IPCL laboratory colonies whose identities are known. The sizes of the DNA ladder bands used for both gel images are indicated. Abbreviations: *Gp (G. pallidipes), Gmm (G. m. morsitans), Gswy (G. swynnertoni), Gff (G. f. fuscipes), Gbr (G. brevipalpis), Gpp (G. p. palpalis), and Gaus (G. austeni).* (TIF 7039 kb)

Additional file 2: **Figure S2.** Sequence alignment of GpSGHV haplotypes in Glossina species: A). Partial alignment of SGHV010 sequences showing the single nucleotide mutations that lead to non-synonymous mutations. Multiple alignment of VNTR-1 (B), VNTR-2 (C), SGHV009 (D), SGHV010 (E) and SGHV038 (F), showing different GpSGHV haplotypes and their phylogenetic relatedness. The positions are based on the reference sequence (GpSGHV-Uga). Abbreviations; H = Haplotype. (TIF 4096 kb)

Additional file 3: **Figure S3.** GpSGHV haplotype network in *Glossina* species: The haplotype network generated based on the ML tree generated based on GpSGHV concatenated sequence of VNTR-1, VNTR-2, ORF9, ORF10 and ORF38. The black dots on the lines represent mutations events between the haplotypes. The different colours represent the *Glossina* species. Abbreviations; KEN (Kenya), TAN (Tanzania), ETH (Ethiopia), ZIM (Zimbabwe), ZAM (Zambia), UGA (Uganda), SA (South Africa), DRC (Democratic republic of Congo), Ruck. (Ruckomechi), BRI-KALRO (Biotechnology Research institute- Kenya agricultural and livestock research organization), GR (Game reserve). (TIF 1390 kb)

## Abbreviations

AW-IPM: Area-wide integrated pest management
GpSGHV: *Glossina pallidipes* salivary gland hypertrophy virus
H: Haplotype
IAPV: Israeli Acute Paralysis Virus
IPCL: Insect Pest Control Laboratory
ITS1: Internal transcribed space 1
MdSGHV: *Musca domestica* salivary gland hypertrophy virus
ML: Maximum likelihood
SGH: Salivary gland hypertrophy
SIT: Sterile insect technique
SNP: Single nucleotide polymorphism
VNTRs: Variable number tandem repeat regions
WNV: West Nile virus
